# Transdermal opioids for cancer pain

**DOI:** 10.1186/1477-7525-4-24

**Published:** 2006-03-31

**Authors:** Tracy L Skaer

**Affiliations:** 1 College of Pharmacy, Washington State University, Wegner Hall Room 105, PO BOX 646510, Pullman, WA 99164-6510, USA

## Abstract

Patients with moderate to severe malignancy-related pain frequently require the use of opioid pharmacotherapy. Unfortunately, many cancer patients continue to be prescribed subtherapeutic doses of pain medications resulting in undo suffering and diminished quality of life. The choice of analgesic pharmacotherapy should be individualized and based on the intensity and etiology of pain reported by the patient. Health care providers must be able to readily quantify the relative analgesic potency when converting from one opioid to another or from one route of administration to another. Transdermal fentanyl is effective and well tolerated pharmacotherapy for the cancer pain patients. However, clinicians need to be cognizant that the U.S./U.K. manufacturer's recommendations for equilalagesic dosing of transdermal fentanyl may result in initial doses that produce subtherapeutic levels and unrelieved pain in some patients. A more aggressive dosing algorithm for transdermal fentanyl using a 2:1 (mg/day of oral morphine: mcg/hr of transdermal fentanyl) conversion ratio that considers both a review of the literature and clinical experience should help clinicians individualize cancer pain pharmacotherapy. Transdermal buprenorphine is now being prescribed in Europe and Australia for chronic and cancer pain management. Buprenorphine's mixed agonist/antagonist activity, dosage ceiling, and high affinity to the opiate receptor limits its use to those patients who do not already require large daily doses of opioids. Thus, buprenorphine may not be an appropriate medication for some patients with advanced unremitting cancer pain.

## Review

Management of malignancy-related pain continues to be a major problem due to undertreatment and/or inadequate selection of adjunctive medications and other modalities combined with opioid therapy. Significant pain is reported in at least one-third of newly diagnosed oncology patients, and 65 to 85 percent of those with advanced disease [1,2]. A broad spectrum of pharmacotherapy is currently available to appropriately manage approximately 90 percent of patients with cancer pain. Unfortunately, many of these patients remain at subtherapeutic doses and continue to experience suboptimal pain control [2-4]. Pain associated with malignancy and its treatment may exacerbate other symptoms associated with cancer including nausea, fatigue, depression, anxiety, weakness, dyspnea, constipation, and impaired cognition [1-6]. Additionally, uncontrolled pain diminishes quality of life and, patients experiencing pain often hesitate in participating in activities of daily living (ADLs) for fear of worsening their pain. Thus, social and family relationships may suffer as this avoidance behavior escalates [6,7].

A thorough pain assessment must be conducted on each patient. This assessment should include pain severity and etiology, age, extent of disease, previously effective and ineffective therapies, concurrent medical problems, and psychosocial status. It is important to note that the care plan for each patient must be individualized, regularly reassessed, and adjusted, if necessary, to maximize pain control and quality of life [3,5,6]. The patient's self-report is very important, as it has been documented that both caregivers and health care workers tend to underestimate pain severity [3,7].

The World Health Organization (WHO) pain management guidelines suggest that the choice of analgesic pharmacotherapy be based on the intensity of pain reported by the patient, not simply its specific etiology [8-10]. Opioids such as morphine, hydromorphone, oxycodone, fentanyl, and buprenorphine, have been shown to be highly effective in alleviating moderate to severe malignant and nonmalignant chronic pain that is not of neuropathic origin [11-14].

### Traditional transdermal dosing conversion

Table 1 provides the manufacturer recommended relative oral morphine to transdermal fentanyl conversion commonly utilized by health care practitioners (HCPs) [15-21]. Patients should be titrated to adequate pain relief with short acting pain medications prior to the initiation of transdermal fentanyl in order to prevent exacerbation of pain or opioid-related adverse effects [22]. Additionally, the manufacturer recommends the following steps to convert patients from oral or parenteral opioids to transdermal fentanyl [20,21]:

**Table 1:** Manufacturer recommended initial fentanyl doses based upon daily oral morphine dose in the US and UK [24]

**24-hour Oral Morphine Dose (mg/day)**	**Transdermal Fentanyl Dose (mcg/hr)**
45–134	25
135–224	50
225–314	75
315–404	100
405–494	125
495–584	150
585–674	175
675–764	200
765–854	225
855–944	250
945–1034	275
1035–1124	300

1. Calculate the previous 24-hour analgesic requirements.

2. Convert this amount to the equianalgesic oral morphine dose.

3. Determine the calculated 24-hour oral morphine dose and corresponding transdermal fentanyl dose.

4. Initiate treatment using this recommended dose, and titrate dosage upward (no more frequently than every 3 days after administering the initial dose or every 6 days thereafter) until analgesic efficacy is attained.

Unfortunately, the aforementioned manufacturer's recommended dosing conversions for transdermal fentanyl provided in Table 1 have been found to underestimate the dosing needs of chronic pain sufferers [22,23]. These subtherapeutic starting doses result in breakthrough pain during initial titration because of failure to increase fentanyl dosages upward in the first 72 hours of therapy. A more aggressive dosing approach is required in the management of malignant pain. Therefore, when titrating the dosage of the transdermal fentanyl, the HCP must consider the daily dose of the immediate release breakthrough pain analgesics required by the patient during the second and third days after initial patch placement and, if necessary, increase the dose of transdermal fentanyl within a 72-hour time span, but at least 18 hours after initiating the currently prescribed patch dose. Moreover, the transdermal fentanyl system (as opposed to injectable fentanyl) has an elimination half life of 13 to 22 hours making it extremely long acting [20]. Thus, it can take as many as 6 days to achieve steady state serum fentanyl concentrations. If the initial starting dose is too low, then the dosage titration necessary to achieve adequate pain control may take even longer [20]. Subtherapeutic pain control is quite distressing for the patient, and can lead to therapy failure and/or discontinuance of pharmacotherapy. This problem is accentuated in patients with chronic pain who have been exposed to opioids previously and therefore require higher doses of these medications to control their pain. Opioid-naïve patients typically need fewer dosage adjustments to reach therapeutic levels.

There have been four case reports in the literature documenting withdrawal syndromes associated with conversion from oral opioids to transdermal fentanyl [24,25]. Withdrawal symptoms in these cases were linked to physiological effects of too low an estimated equianagesic starting dose and not related to psychological dependence. Therapeutic fentanyl levels can take 12 to 18 hours to occur after initial patch application. Thus, patients at greatest risk for withdrawal are those who are physiologically dependent and stop taking their oral opioid medication prior to the first application of the transdermal patch and/or prior to the achievement of a steady-state fentanyl serum level. Those patients who are started on a subtherapeutic dose of transdermal fentanyl at the time of dose conversion from opioid pharmacotherapy are also at risk of experiencing withdrawal symptoms and breakthrough pain.

### Dosing algorithm for transdermal fentanyl

**Figure 1:**
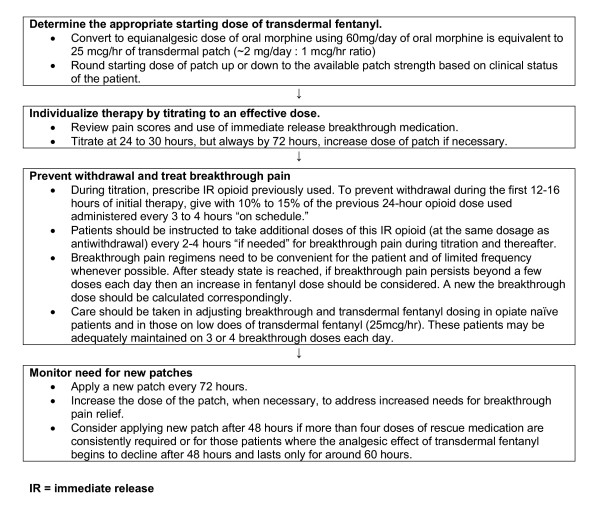
Dosing algorithm for transdermal fentanyl in the cancer patient [adapted from Breitbart et al 28].

**Figure 2:**
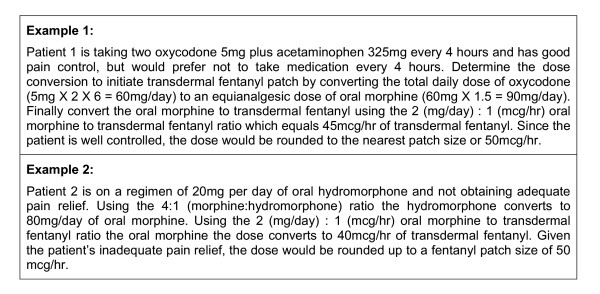
Examples of determining the appropriate initial fentanyl patch size [adapted from Breitbart et al 28].

Cancer patients require an aggressive approach to their pain management and the initial dosing transdermal fentanyl. Care must be taken to avoid subtherapeutic dosing that can compromise patient care and result in uncontrolled pain during the initial conversion and titration period. The conversion table supplied by the manufacturer in Germany (Table 2) is much less conservative than the US/UK table presented in Table 1[20,21,26]. The German conversion rate of approximately 2(mg/day):1(mcg/hr) (60 mg per day of oral morphine is equilanalgesic to 25 mcg per hour of transdermal fentanyl) best translates into an optimal initial starting dose for the cancer patient [26]. With this in mind, Figure 1 provides a dosing algorithm for this purpose and Table 3 extrapolates the recommended dose conversion to transdermal fentanyl from morphine and other commonly employed opioids for moderate to severe pain [15,26]. Once an approximate starting dose is calculated, round up or down to the available patch strength (25 mcg/hr, 50 mcg/hr, 75 mcg/hr, 100 mcg/hr) based on the clinical status of the patient. If the patient has adequate pain relief from their currently prescribed pain pharmacotherapy, it is recommended that the calculated dose be rounded to the nearest patch size (see Figure 2: Example 1). However, it the patient is experiencing pain at the time of conversion then the dose should be rounded up to the nearest patch strength (see Figure 2: Example 2).

**Table 2:** Recommended initial fentanyl doses based upon daily oral morphine dose in Germany [20,26]

**24-hour Oral Morphine Dose (mg/day)**	**Transdermal Fentanyl Dose (mcg/hr)**
0–90	25
91–150	50
151–210	75
211–270	100
Every additional 60 mg	25

**Table 3:** Recommended dose conversion to fentanyl from other selected opioids [15,28]

**Transdermal Fentanyl (ncg/hr)**	**Morphine (mg/day) IM PO**		**Oxycodone (mg/day) IM PO**		**Hydromorphone (mg/day) IM PO**	
25	20	60	NA	40	3	15
50	40	120	NA	80	6	30
75	60	180	NA	120	9	45
100	80	240	NA	160	12	60

IM = intramuscular; NA = not applicable; PO = oral

A multi-center trial conducted by Donner et al supports the safety and efficacy of the German recommended 2(mg/day):1(mcg/hr) oral morphine to transdermal fentanyl ratio [23]. The study involved 98 patients with cancer-associated pain who were converted directly from sustained release oral morphine to transdermal fentanyl. The initial fentanyl dose was calculated by the dose of sustained-release morphine prescribed to the patient prior to enrollment into the study. The 2(mg/day):1(mcg/hr) conversion ratio was employed. Breakthrough pain relief was provided to the patients in the trial through the use of supplemental immediate release liquid morphine as needed. Pain relief with transdermal fentanyl was similar to that of sustained release morphine, but the use of supplemental liquid morphine for breakthrough pain was significantly higher for those patients receiving transdermal fentanyl. Constipation was less problematic in patients treated with fentanyl. There was no significant difference in vital signs and adverse effects between the two groups. Respiratory depression was not seen; however three patients experienced morphine withdrawal symptoms within the first 24 hours of transdermal fentanyl therapy. The highest dose of transdermal fentanyl administered was 500 mcg per hour.

A more recent study of 1,828 cancer patients who were either opioid naïve, taking codeine or morphine for their pain used an average 3(mg/day):1(mcg/hr) conversion ratio for enrollment into each study group [27]. The results showed that the 3:1 ratio selected for the initial dose of transdermal fentanyl was too conservative and all patients required an escalation in dose to the 2(mg/day):1(mcg/hr) ratio in the first 48-hours of initial fentanyl therapy. The most common side effect of the study was constipation with an averaged incidence across the study groups of 16.6%. The most severe adverse effect of transdermal fentanyl was nausea but the incidence was low (1.4%).

### Dosage titration and breakthrough pain

Evaluations as to whether the initial starting dose of transdermal fentanyl is providing adequate pain relief should be conducted during the first 72 hours after initiation. If the patient requires more than two doses of breakthrough medication over a 24-hour period for adequate pain relief, than consideration should be given to increase the fentanyl patch dose. At low doses of opioids, the patch is normally increased in 25 mcg per hour increments. It may be increased in increments of 50 mcg per hour if the severity of the pain, number of breakthrough doses required, and total dose of transdermal fentanyl needed for adequate relief warrants this level of increase. Fifty (50) mcg incremental dose increases should only be employed in patients that are not opioid naïve and where a 50 mcg escalation represents and appropriate percentage of the entire daily opioid dose. The optimal dose of transdermal fentanyl should be based on an ongoing evaluation of the level of pain relief achieved and the amount of breakthrough medications utilized. It is important to note that it can take from 12 to 18 hours to reach a clinical relevant serum level after initial patch placement. Consistent serum levels are achieved after 16 to 20 hours, and steady state is attained at about 72 hours [20].

Upon initial dosage titration, and in order to minimize the risk of opioid withdrawal during the first 16 hours of transdermal fentanyl therapy, patients should be instructed to take the prescribed immediate release opioid every 3 to 4 hours [28]. The dose of "anti-withdrawal" medication should be equal to 10 to 15 percent of the total daily dose of opioid that the patient received prior to the start of fentanyl pharmacotherapy. As an example, if the patient is applying a 50 mcg per hour fentanyl patch every 72 hours, the equivalent daily oral dose of oral immediate release oxycodone is approximately 80mg. Thus, the patient would be prescribed at least 8 to 12 mg (10 to 15% of 80 mg) of oxycodone every 3 to 4 hours (not as needed) for the first 12-16 hours of transdermal fentanyl pharmacotherapy, and thereafter only as needed. Some patients may require additional immediate release doses as frequently as every 2 hours as needed for breakthrough pain. Thus, the use of "anti-withdrawal" medication must be differentiated from breakthrough pain medication, since the patient mentioned above should take at least 8 mg of oxycodone every 4 hours to abate development of withdrawal symptoms during this first 16 hour phase regardless of whether breakthrough medication is required during that same time period.

Breakthrough pain should be treated with medications that are simple to administer, offer rapid pain relief, and have a reasonably short half-life [28]. Immediate release morphine, hydrocodone, or oxycodone is commonly used for this purpose [28]. Patients can continue to take the short-acting opioid that was previously effective for breakthrough pain [28,29]. Doses of immediate release pharmacotherapy for breakthrough pain commonly utilized are 10 to 15 percent of the previous total daily opioid dose given every 2-4 hours on an "as needed" basis [29]. Ideally patients should not take more than two doses of immediate release breakthrough medication each day once a steady state serum concentration of fentanyl has been reached [28,29]. If the breakthrough pain is persistent and requires more than two doses of immediate release medication during a 24-hour period, then consideration may be given to increasing the transdermal fentanyl dose. At lower transdermal fentanyl doses of 25mcg/hour, however, up to 4 doses of breakthrough medication may be acceptable without increasing the transdermal patch dose. For example, if a patient is receiving fentanyl 25mcg/hr with a daily oxycodone PRN breakthrough dose of 5mg PO 3-4 times per day, it may be too soon to increase the fentanyl dose, since the next available transdermal patch size is 50mcg, or twice the current dose. Comparatively, if the patient is requiring just 3-4 doses of oxycodone per day (15-20mg), doubling the fentanyl dose would result in the patient receiving an approximately 40mg oxycodone increase per day when in fact only 15-20mg per day was already adequate. Patients who require an increase in the dose of their around-the-clock sustained release pharmacotherapy (transdermal fentanyl) as a result of disease progression or other factors should be given an equivalent increase in the dose of the breakthrough pain medication.

### Patch application considerations

Good adhesion of the fentanyl patch to the skin is essential for maximum efficacy, therefore patients must be instructed on the proper technique for patch application [30]. Hair on the skin should be clipped, not shaved, in order to avoid abrasions where the patch is to be applied. This skin should be clean, dry, and undamaged. Soap and/or topical alcohol-based products should not be used to cleanse the area immediately prior to patch placement; water only should be used and the area must be completely dried. After removal of the plastic backing, the patch should be held firmly in place for about 30 seconds. A finger should be run around the edge of the patch to ensure that adhesion has occurred around all edges. The top of the patch should be rubbed for approximately 3 minutes. The TTS Multicentre Study Group reported that 82% of patients had no problems with patch adherence when appropriate technique was employed [31]. There are some instances where additional adhesion with tape may be needed especially in warm weather or in patients who are diaphoretic. Use of an occlusive dressing may be helpful if tape is insufficient. Patients should also be instructed to rotate sites when changing patches in order to minimize changes in serum levels due to build up of subcutaneous depots, and to minimize skin irritation [30].

For the majority of patients, the analgesic effect of fentanyl will last for 72 hours and a new patch applied after that time. Changing fentanyl patches more often than every 48 hours is not recommended. Many clinicians recommend increasing the dosage rather than shortening the dosing interval. However some patients may find that the effect begins to decline after 48 hours and lasts only for around 60 hours [32,33]. In these instances, the clinician should seriously consider changing the fentanyl patch every 48 hours.

### Safety, tolerability, and contraindications

The overall tolerability of transdermal fentanyl is very good. The most frequently observed adverse effects include nausea, vomiting and constipation [20,34,35]. Constipation and somnolence occur significantly less frequently with transdermal fentanyl as compared to sustained release oral morphine (constipation: 17% vs. 48%, respectively, p < 0.001; somnolence: 13% vs. 25%, respectively, p < 0.001) [34,35]. Nausea and vomiting is significantly more common with sustained release morphine than with transdermal fentanyl (31% vs. 28%, respectively, p < 0.001). Skin reactions (i.e. rash, and application site reactions – erythema, papules, itching, and edema) in cancer patients have been reported at a frequency between 1 and 2 percent [20]. Opioid withdrawal symptoms (e.g. nausea, vomiting, diarrhea, anxiety, and shivering) may occur in some patients after discontinuation of transdermal fentanyl, conversion to another opioid, or after lowering the fentanyl dosage [21].

Hypoventilation (defined as respiratory rates of less than 8 breaths per minute or a pC02 greater than 55mm Hg) was reported in three (2%) of the 153 patients with cancer pain during the pre-marketing trial [21]. However, clinical relevant fentanyl-induced respiratory depression in chronic pain patients was not observed in several clinical trials [27,34-37]. Serious or life-threatening hypoventilation has been documented on opioid-naïve patients and in postoperative setting. Transdermal fentanyl is therefore contraindicated in the management of acute or postoperative pain and intermittent, mild pain which can be adequately managed with other pharmacotherapy [21,22]. Transdermal fentanyl should not be administered to children under the age of 12 or patients under 18 years old who weigh less than 50 killigrams [21]. Those with are hypersensitive to either fentanyl, other phenylpiperidines (i.e. merperidine, sufentanil, remifentanl, alfentanil), or the adhesives used in the system should also not receive this medication [21].

Patients should be instructed to refrain from driving or operating machinery immediately following the initiation of transdermal fentanyl, or after any dosage increase [21,22]. Patients should be warned against the use of electric blankets, heating pads, hot tubs, saunas, and heat lamps while wearing transdermal fentanyl patches. The heat produced by these items can potentially increase the amount of fentanyl released from the system [21]. Additionally, fever may enhance fentanyl absorption [21,34,35]. Therefore, patients who are febrile need to be monitored for enhanced pharmacological effects and their dosage adjusted if necessary.

Concomitant use of other centrally acting depressants such as sedatives, other opoids, hypnotics, phenothiazines, tranquillizers, skeletal muscle relaxants, anesthetics, sedating antihistamines, and alcohol can cause hypoventilation, acute sedation, or hypotension in patients taking transdermal fentanyl [21]. It is advisable to reduce the dosages of one or all of these agents when polytherapy of this nature is considered [21]. Finally, the transdermal fentanyl reservoir system should not be cut or damaged, as the integrity of the transdermal system is destroyed. Safe disposal of the used transdermal fentanyl systems is important in order to avoid diversion, accidental poisoning of infants, children, animals, and adults [21].

### Transdermal buprenorphine

Buprenorphine, a centrally acting opioid analgesic, is now being prescribed in Europe and Australia for chronic and cancer pain management [38-44]. Buprenorphine is a synthetic opioid which is lipophilic, water soluble, and has a low molecular weight; these properties allow for tissue penetration and make it suitable for transdermal delivery [38,39]. The buprenorphine is contained in a matrix patch that is applied to the skin for a three-day duration. The matrix patch differs from the reservoir patch technology. In a matrix system, the substance is an integral part of the polymer structure of the patch rendering the buprenorphine patch more robust in handling. While damaging a reservoir patch might result in "dose-dumping" and potentially overdosing the patient, damaging a matrix patch does not necessarily interfere with the controlled release of the medication [38,39].

Transdermal buprenorphine is available with release rates of 35, 52.5, and 70 micrograms per hour which corresponds to daily doses of 0.8, 1.2, and 1.6 milligrams of buprenorphine, or approximately 60, 90, and 120 milligrams per day equivalent of oral morphine, respectively [38-41,44]. Steady state serum concentration of buprenorphine can take several days to achieve with the transdermal formulation. The terminal half-life when delivering buprenorphine by the transdermal system has been reported as 25 to 27 hours [38-40,44]. A clinically effective or analgesia producing serum concentration is reached in about 12 hours [38-40,44]. Therefore, like transdermal fentanyl, it is again important to provide immediate release opioid medication to assist in the prevention of withdrawal symptoms during initial dosage titration and for treatment of breakthrough pain. However, in the case of buprenorphine, a less conservative breakthrough dose regimen may be acceptable, since the antagonist activity of buprenorphine will help to avoid additive opioid-induced somnolence.

Buprenorphine TDS has been shown to be quite effective against chronic, severe pain in three multicenter, randomized, double-blind, placebo-controlled studies [45-47]. Patients enrolled in these studies had moderate to severe or severe to very severe chronic pain of malignant or nonmalignant origin. In patients who were unsuccessfully treated with weak opioids or morphine, 36.6% and 47.5% of buprenorphine 35 mcg/hour and 52.5 mcg/hour recipients, respectively, experienced at least satisfactory analgesia and received ≤ 0.2 mg/day of sublingual buprenorphine compared with 16.2% response rate for those receiving placebo (p ≤ 0.05) [45]. The requirement for breakthrough medication was reduced from baseline by approximately 50-70% in patients treated with transdermal buprenorphine [45-47]. Those receiving transdermal buprenorphine experienced greater pain relief, reduced pain intensity and longer pain-free sleep [45-47]. A more recent, multicenter, open-labeled, uncontrolled, prospective, observational clinical practice study involving 1,223 patients with moderate to severe chronic pain demonstrated that transdermal buprenorphine was effective in alleviating cancer and non-cancer pain and was overall well tolerated [48]. These patients also experienced a significant improvement (p < 0.001) in quality of life scores and reported very good to good pain relief (p < 0.001).

Transdermal delivery of buprenorphine provides for a slower increase in serum concentration and no peak-and-trough effects as seen with the sublingual route of administration. As a result, there are fewer adverse events reported when using the transdermal delivery system for this medication [38-44]. Transdermal buprenorphine was usually well tolerated and adverse events reported in clinical trials were generally mild to moderate in severity. Side effects included local erythema 26.6%, local pruritis 23.2%, nausea 16.7%, vomiting 9.3%, dizziness 6.8%, sedation 5.6%, constipation 5.3%, and erythema 4% [38,45,46]. Adverse events could generally be attributed to either local skin reactions at the application site, buprenorphine (systemic events common to opioid administration), or underlying disease. Adverse events were more frequently reported in patients with malignant pain than those without (46.6% vs. 34.2%, respectively). Transdermal buprenorphine was associated with a low rate of withdrawals due to adverse events [38,45,46]. In one study, only 10.8% of the patients withdrew because of adverse events during a 15-day treatment period [45].

Of very important note is that because buprenorphine is a mixed opioid agonist/antagonist, it does have a dosage ceiling [38,39]. Therefore, those patients who are already on large does of chronic opioids (e.g. 300 mg or more of oral morphine per day) are not considered appropriate candidates for transdermal burprenorphine therapy. Moreover, these patients would be at significant risk of opioid withdrawal because buprenorphine's affinity for the opiate receptor is higher than morphine, and the overall opiate agonist activity would not be adequate to overcome the withdrawal symptoms that will otherwise be seen [38,39]. Finally, transdermal buprenorphine should not be used in opioid-dependent individuals undergoing treatment for narcotic withdrawal [39].

Based on the currently available clinical trial data, transdermal buprenorphine is a valuable alternative to other available opioids in many chronic pain conditions. However, despite the positive data presented in these four clinical trials, more controlled studies are needed to determine the place of transdermal buprenorphine among current treatment strategies for chronic and cancer pain, and to explore if and whether transdermal buprenorphine would be of any value in the treatment of difficult pain conditions such as neuropathic pain.

## Conclusion and recommendations

Many pharmacotherapeutic choices are available for the management of cancer pain. HCPs must be able to readily quantify the approximate relative analgesic potency when converting from one opioid to another and from one route to another. Transdermal formulations of fentanyl and burprenorphine are very useful pharmacotherapy for the cancer patient experiencing moderate to severe pain. Buprenorphine's mixed agonist/antagonist activity, dosage ceiling, and high affinity to the opiate receptor limits its use to those patients who do not already require large daily doses of opioids. Thus, buprenorphine may not be an appropriate medication for some patients with advanced unremitting cancer pain. Clinicians need to be aware that the relative opioid conversion tables commonly utilized are often based on the results of single-dose studies and frequently underestimate the dosage required for the cancer pain patient. Generally, a more aggressive approach to converting a patient to transdermal fentanyl may be warranted in the cancer patient. Care must be taken to individualize each patient's pain management in order to prevent opioid withdrawal and substantially reduce the undertreatment and overtreatment of cancer-related pain and its associated negative impact on patients' quality of life.
